# Residential Proximity to Major Roadways Is Not Associated with Cardiac Function in African Americans: Results from the Jackson Heart Study

**DOI:** 10.3390/ijerph13060581

**Published:** 2016-06-13

**Authors:** Anne M. Weaver, Gregory A. Wellenius, Wen-Chih Wu, DeMarc A. Hickson, Masoor Kamalesh, Yi Wang

**Affiliations:** 1 Richard M. Fairbanks School of Public Health, Indiana University, Indianapolis, IN 46202, USA; yw54@iu.edu; 2 School of Public Health, Brown University, Providence, RI 02903, USA; gregory_wellenius@brown.edu (G.A.W.); wen-chih_wu@brown.edu (W.-C.W.); 3 School of Public Health, Jackson State University, Jackson, MS 39217, USA; demarc.a.hickson@jsums.edu; 4 Richard L. Roudebush VA Medical Center, Indianapolis, IN 46202, USA; Masoor.Kamalesh@va.gov

**Keywords:** ambient air pollution, distance to road, cardiac function, African Americans, ejection fraction, E-wave velocity, isovolumic relaxation time, left atrial diameter index, pulmonary artery systolic pressure

## Abstract

Cardiovascular disease (CVD), including heart failure, is a major cause of morbidity and mortality, particularly among African Americans. Exposure to ambient air pollution, such as that produced by vehicular traffic, is believed to be associated with heart failure, possibly by impairing cardiac function. We evaluated the cross-sectional association between residential proximity to major roads, a marker of long-term exposure to traffic-related pollution, and echocardiographic indicators of left and pulmonary vascular function in African Americans enrolled in the Jackson Heart Study (JHS): left ventricular ejection fraction, E-wave velocity, isovolumic relaxation time, left atrial diameter index, and pulmonary artery systolic pressure. We examined these associations using multivariable linear or logistic regression, adjusting for potential confounders. Of 4866 participants at study enrollment, 106 lived <150 m, 159 lived 150–299 m, 1161 lived 300–999 m, and 3440 lived ≥1000 m from a major roadway. We did not observe any associations between residential distance to major roads and these markers of cardiac function. Results were similar with additional adjustment for diabetes and hypertension, when considering varying definitions of major roadways, or when limiting analyses to those free from cardiovascular disease at baseline. Overall, we observed little evidence that residential proximity to major roads was associated with cardiac function among African Americans.

## 1. Introduction

Despite progress in the treatment and prevention of cardiovascular disease (CVD), it remains the leading cause of death in the U.S. Heart failure (HF), in particular, remains a common cause of morbidity and mortality. Although mortality from all forms of CVD combined has decreased by nearly one third from 1999 to 2009, the number of deaths from HF in 2009 was approximately the same as it was in 1995 [1]. HF is a syndrome in which the heart is incapable of maintaining cardiac output adequate to meet metabolic requirements and accommodate venous return [2]. HF can be caused by impaired cardiac function [2], indicated by the following echocardiographic parameters: depressed (<50%) left ventricular ejection fraction (LVEF) (a marker of systolic dysfunction), lower E-wave velocity, longer isovolumic relaxation time, larger left atrial (LA) diameter index (markers of diastolic dysfunction), and pulmonary artery systolic pressure (PASP) (a marker of pulmonary vascular function).

One documented risk factor for CVD in general and HF in particular is exposure to ambient air pollution, including traffic-related air pollution [3]. Long-term exposure to traffic-related air pollutants, such as fine particulate matter (PM_2.5_), nitrogen dioxide (NO_2_), and black smoke, has been shown to be associated with increased incidence [4,5], and mortality from heart failure [4]. Residential proximity to major roadways is a proxy measure of long-term exposure to traffic-related air pollution and has been shown to increase cardiovascular morbidity [3,6,7]. However, results from studies of exposure to traffic-related air pollution and cardiac function have been inconsistent. In an experimental study in mice, long-term exposure to PM_2.5_ was shown to be associated with poorer cardiac function, including decreased ratio of E-wave to A-wave velocity (a marker of diastolic function) [8]. However, in an observational study in humans, Van Hee *et al.* failed to find an association between residential proximity to roads and LV ejection fraction (a marker of systolic function) [9]. In another observational study in humans, Leary *et al.* found associations between NO_2_ concentrations and right ventricular mass and right ventricular end-diastolic volume, but not right ventricular ejection fraction (markers of right-sided heart dysfunction) [10]. The associations between traffic-related pollution and markers of cardiac function remain unclear.

African Americans are at an increased risk of all-cause CVD and HF incidence and mortality compared to European Americans [1,11,12,13]. The high CVD incidence and mortality among African Americans could be at least partially explained by a higher prevalence of traditional cardiovascular risk factors, such as diabetes mellitus and hypertension [1]. The state of Mississippi has the highest proportion of African Americans and the highest rate of death from cardiovascular disease of any state in the U.S. [1,14]. In this study, we evaluated the effects of living close to major roadways on indicators of cardiac function among African Americans residing in Mississippi who were participants of the Jackson Heart Study (JHS).

## 2. Materials and Methods

Between 2000 and 2004, JHS recruited a total of 5301 non-institutionalized African American men and women aged 21 years and older, residing in the tri-county Jackson, Mississippi Metropolitan Statistical Area (MSA). JHS recruited participants by random sampling of the Atherosclerosis Risk in Community (ARIC) Study Jackson subpopulation (22%), random sampling of eligible Jackson MSA households (17%), structured community-based volunteer programs (30%), and recruitment of eligible family members (31%), as previously described [15,16]. The JHS cohort has been shown to be representative of the underlying African American population living in the Jackson MSA [17]. Upon enrollment, participants completed an in-home interview followed by a clinic visit [15,16]. All participants provided written informed consent. All JHS protocols were reviewed and approved by the Institutional Review Boards at Jackson State University, Tougaloo College, and the University of Mississippi Medical Center (UMMC IRB file #1998-6004). This analysis was approved by the Institutional Review Board at Indiana University (Protocol #1311707518).

### 2.1. Residential Proximity to Major Roadways

In Jackson, MS, the main source of several pollutants, including ozone and NO_2_, has been shown to be from mobile sources (traffic), although there was some contribution to nitrous acid concentrations from stationary combustion (industries) in the central and northeast regions of the city [18]. However, there are few major industrial sources of air pollution. Therefore, we believe that long-term exposure to traffic pollution is an appropriate proxy measure for exposure to long-term air pollution among those living in and near Jackson. We used residential distance to the nearest major roadway as marker of long-term exposure to traffic-related air pollution. We used ArcGIS (version 9.2; ESRI Inc., Redlands, CA, USA) to geocode participants’ addresses and calculate the straight-line distance from each residence to the nearest major roadway. We defined major roadways as a road with U.S. Census Feature Class Code A1 (primary highway with limited access) or A2 (primary road without limited access), as previously described [19,20,21].

### 2.2. Markers of Cardiac Function

During the baseline clinic visit (2000–2004), JHS participants underwent prioritized 30-minute two-dimensional and M-mode echocardiography with parasternal, apical, and subcostal windows [16]. Echocardiography was performed using a Sonos 4500 echocardiograph (Philips Medical Systems, Andover, MA, USA) by a certified, experienced cardiac ultrasonagraphy technician [16,22]. All echocardiograms were analyzed using the American Society of Echocardiography conventions [22,23]. We examined several markers of cardiac function as outcomes. A marker of systolic function was left ventricular ejection fraction (LVEF); markers of diastolic dysfunction were E-wave velocity (m/sec), LV isovolumic relaxation time (msec), and left atrial (LA) diameter index (mm/m^2^); and a marker of pulmonary vascular function was pulmonary artery systolic pressure (PASP). All markers of cardiac function were determined by M-mode echocardiography, with the exception of LA diameter index. LA diameter index was determined by two-dimensional echocardiography due to limitations in visualizing the left atrium using M-mode echocardiography. LVEF was defined as the fraction of change in left ventricular volume between systole and diastole divided by the total ventricular volume in diastole, estimated to the nearest 5% [24]. For this analysis, we classified LVEF as normal (≥50%) or depressed (<50%) [25,26,27]. Quantitative Doppler data were used to measure peak E-wave velocity [16,22]. LA diameter index was defined as the LA end-systolic diameter (mm) divided by body surface area (m^2^) [28]. PASP was defined as the tricuspid regurgitant peak velocity, for those with detectable tricuspid regurgitation, plus right atrial pressure, assumed to be 5 mmHg [26,29].

### 2.3. Covariates

At baseline, participants were given an in-person interview, querying demographics, health history, lifestyle factors, and healthcare access. Shortly thereafter, participants were given a clinical exam. Details of the baseline interview and exam are described elsewhere [16]. During the clinical exam, medical history was assessed by interview, anthropometrics were measured, sitting blood pressure was measured, and blood samples were drawn and analyzed. Participants brought all current medications, which were inventoried by study staff.

Potential covariates were derived from physical measurements taken at the baseline clinical exam and responses given during the baseline interview. The following variables were derived from physical measurements conducted during the baseline clinical exam: body mass index (BMI) was calculated from weight (kg) divided by height (m) squared and evaluated as a continuous variable; serum 25-OH vitamin D3 and sodium were assessed based on blood samples obtained during the first JHS clinic visit. The following variables were derived from the baseline interview: age in years (continuous), sex (male/female) smoking status (never, former, current), and alcohol consumption in the past 12 months (yes/no). Nutritional status and physical activity were categorized as poor, intermediate, or ideal according to Life’s Simple 7 criteria [30,31]. Education was categorized as the highest level of education completed: less than high school, high school or GED, college degree or certificate, and graduate or professional school. Type of insurance was categorized as none, private only, public only, and both public and private. Occupation was categorized based on Sims *et al.* [32] and the distribution in our sample as: management/professional, service, sales, and other. Household income level was determined based on self-reported income, family size, number of children <18 years old, and the U.S. Census designated poverty level for the year of data collection; participants were classified into one of four income levels: low, lower-middle, upper-middle, or high [33]. Markers of neighborhood socioeconomic status (NSES) were assessed at the census tract level based on six variables: percent of adults aged >25 years with less than high school education, percent of males who were unemployed, percent of households with income below the poverty line, percent of households receiving public assistance, percent of households with children headed by a woman, and median household income, as described in Dubowitz *et al.* [34]. NSES was then converted to a z-score, as described by Diez Roux *et al.* and was evaluated as a continuous variable [35].

Participants were classified as hypertensive if their supine blood pressure at the baseline clinical examination was ≥140/90 mmHg or they used blood pressure lowering medication (self-report or medication inventory) [36,37]. Participants were classified as having diabetes if they used any diabetes medications (self-report or medication inventory), measured hemoglobin A1c levels were ≥6.5%, or fasting glucose measurement was ≥126 mg/dL [38]. Participants were classified having hyperlipidemia if total cholesterol was ≥240 mg/dL or low-density lipoprotein cholesterol level was ≥160 mg/dL or they reported use of lipid-lowering medications (self-report or medication inventory) [1].

### 2.4. Statistical Analyses

We limited our analyses to participants (91.8%) with valid geocoded addresses at the street level and those with valid echocardiography data. Since many (*n* = 214) participants were missing all echocardiographic data, we compared descriptive characteristics among those missing and not missing all outcome measures at baseline. Many participants (35.2%) were missing PASP, as they did not have detectable tricuspid regurgitation. These participants were included in the main analytic data set.

We categorized residential distance to A1 or A2 roads as <150 m, 150–299 m, 300–999 m, and ≥1000 m [39]. We chose cutpoints of 150 and 300 m because particulate matter concentration from highway traffic pollution has been shown to decrease by 50% at 150 m and fade into background levels after 300 m, and these cutpoints have been used in our previous studies [39,40]. We chose to include an additional cutpoint of 1000 m so our results may be comparable to reference categories in several previous papers [6,19,20,21]. Additionally, we examined residential distance to A1 or A2 roads as a continuous variable; due to the distribution in the dataset, we present results with log-transformed distance to A1 or A2 roads as an exposure of interest.

We estimated the odds of depressed LVEF among those who lived <150 m, 150–299 m, and 300–999 m compared to those who live ≥1000 m from A1 or A2 roads, as well as the odds of depressed LVEF associated with log-transformed continuous residential distance to A1 or A2 roads using multivariable logistic regression. We estimated the differences in E-wave velocity, log isovolumic relaxation time, LA diameter index, and log PASP among those who live <150 m, 150–299 m, and 300–999 m compared to those who live ≥1000 m from A1 or A2 roads, as well as differences in the above indices of cardiac function associated with log-transformed continuous residential distance to A1 or A2 roads using multivariable linear regression. We examined several parameterizations of isovolumic relaxation time and PASP; due to their distribution, to meet linear modeling assumptions, we examined these variables as log-transformed variables.

We analyzed four models: unadjusted models; minimally-adjusted models, adjusted for variables decided *a priori* (age, sex, BMI, alcohol consumption, and physical activity status); fully adjusted models, adjusted for *a priori* covariates plus additional covariates that were associated with exposure and outcome at a *p*-value of 0.05 and were not collinear with other covariates (variance inflation factor <10) (education, occupation, NSES, smoking status); and models adjusted for all covariates in the fully adjusted model plus diabetes and hypertension. Hypertension and diabetes are important CVD risk factors and both may also be associated with long-term exposure to traffic pollution [41,42,43,44]. However, it is unclear whether these risk factors should be treated as confounders or whether they reflect potential mediators of the association between roadway proximity and cardiac function. Thus, we considered both possibilities. Although associated with exposure at the *p* = 0.05 level, we did not adjust for household income status due to a great number of participants (*n* = 462) with missing values. Sensitivity analyses showed additional adjustment for household income did not substantially change estimates of association.

### 2.5. Sensitivity Analysis

We conducted additional sensitivity analyses. First, we redefined the exposure (residential distance to major roadway) as residential distance to A1 (primary highway with limited access) roads as A1 roads may be busier and have more diesel traffic compared to A2 roads. We classified residential distance to A1 roads as <150 m, 150–299 m, 300–999 m, and ≥1000 m, as well as log-transformed distance as a continuous variable, similar to primary analyses, adjusting for the same covariates as in primary analyses. Second, given the possibility that our chosen cutpoints of distance to road may be inappropriate for the relatively small number of participants living nearest to major roads, we also re-classified distance to A1 or A2 roads as <250 m *vs.* ≥250 m, <300 m *vs.* ≥300 m, and <500 m *vs.* ≥500 m. Third, we excluded JHS participants with CVD at baseline. CVD was defined as having a history of myocardial infarction (based on self-report or electrocardiogram), a history of carotid angioplasty (self-report), and/or a history of stroke (based on self-report of doctor diagnosis or experiencing any of following symptoms with sudden onset and lasting at least 24 h: loss of speech, loss of vision, double vision, numbness or tingling, paralysis or weakness, or dizziness or loss of balance. Fourth, we limited our analyses to those who reside in urban areas, defined by 2010 U.S. Census Urban and Rural Classification. Finally, we examined models with several parameterizations of outcomes, including: LVEF as a semi-continuous (to nearest 5%) variable and in categories of <40%, 40%–55%, >55%; isovolumic relaxation time as continuous (not log-transformed) and categorical, split at the median; and PASP as continuous (not log-transformed). Additionally, we plotted the spline associations with log-transformed distance to A1 or A2 roads for E-wave velocity, log isovolumic relaxation time, LA diameter index, and log PASP, adjusting for covariates in the fully-adjusted model using R 3.1.0 (The R Foundation for Statistical Computing, Vienna, Austria). All other analyses were conducted using SAS version 9.4 (SAS Institute Inc., Cary, NC, USA); results were considered statistically significant at a two-sided *p*-value of <0.05.

## 3. Results

The Jackson Heart Study recruited a total of 5301 participants. We excluded 214 participants who were missing data on all outcomes, 211 participants whose addresses were not geocoded at baseline, and 10 participants with severe mitral regurgitation or aortic leaflets, making our final sample size 4866 participants. We examined descriptive characteristics among those missing (*n* = 214) and not missing (*n* = 4866) all measures of cardiac function. Those missing all measures of cardiac function were younger (53.2 *vs.* 55.5 years), more likely to drink alcohol (51.8% *vs.* 44.2%), more likely to be current smokers (18.5% *vs.* 12.9%), and more likely to have less than high school education (25.8% *vs.* 19.9%).

Of the 4866 participants in our analytic sample, 106 (2.2%) lived <150 m, 159 (3.3%) lived 150–299 m, 1161 (23.9%) lived 300–999 m, and 3440 (70.7%) lived ≥1000 m from A1 or A2 roads (Table 1). Age, sex, and BMI were similar among all categories of distance to major roadways; mean age was between 54 and 59 years, over 60% of participants were female, and mean BMI was obese (≥30 kg/m^2^).

Those residing closest to A1 or A2 roads were most likely to be have poorer socioeconomic indicators, including education, income, and lower proportion in management or professional occupations (*p* < 0.05). Those living closest to A1 or A2 roads also had the lowest prevalence of hypertension (55.8%) (*p* = 0.005). Less than half of participants in each category of residential distance to A1 or A2 roads consumed alcohol in the past year. There were no differences in diabetes or hyperlipidemia prevalence by distance to A1 or A2 roads. In bivariate analyses, there were no statistically significant differences in indicators of cardiac function by residential distance to A1 roads.

We did not observe any statistically significant differences in markers of cardiac function between those who live closest and furthest to A1 or A2 roads (Table 2). Since results were similar in unadjusted, minimally-adjusted and fully-adjusted models, we only present results from fully-adjusted models in Table 2. Compared to those who lived ≥1000 m from A1 or A2 roads, point estimates indicate those who lived <150 m had lower odds of having depressed LVEF, lower E-wave velocity, longer log isovolumic relaxation time, greater LA diameter index, and lower log PASP; however, none of these results were statistically significant. Those who lived 150–299 m from A1 or A2 roads had a 0.4 mm/m^2^ lower LA diameter index (95% CI −0.7, −0.02) compared to those who lived ≥1000 m. Those who lived 300–999 m from A1 or A2 roads had higher log PASP compared to those who lived ≥1000 m (0.02, 95% CI 0.001, 0.04). Results were similar after additional adjustment for hypertension and diabetes.

In sensitivity analyses, when observing residential distance to A1 roads as an exposure of interest, in unadjusted models, we observed that those who lived 300–999 m from an A1 road had a −0.3 mm/m^2^ smaller LA diameter index compared to those who lived ≥1000 m from an A1 road and distance from and A1 road was inversely associated with log PASP; however, these associations were attenuated after adjustment. After adjustment, compared to those who lived ≥1000 m from an A1 road, we observed that those who lived 300−999 m from an A1 road had a 51% lower odds of having a depressed LVEF (95% CI −72%, −14%); those who lived <150 m from a road had a 139% greater odds of having depressed LVEF, but this was not statistically significant (Table S1). Similar to our main analyses, we did not observe any other associations between markers of cardiac function and distance to A1 roads. We did not observe any associations when examining distance to A1 or A2 roads with cutpoints at 250 m, 300 m, or 500 m. When excluding 513 participants with CVD at baseline, those who lived 150–299 m from an A1 or A2 road had a 0.4 mm/m^2^ (95% CI −0.8, −0.03) smaller LA diameter index and LA diameter index was associated with log-transformed distance to A1 or A2 road (0.07, 95% CI 0.01, 0.1) (Table S2). When limiting to 3647 participants who live in urban areas (results not shown), results were not substantially different from our main analysis. Likewise, results were similar with alternate parameterizations of LVEF, isovolumic relaxation time, and PASP. Spline plots showed that the associations between log-transformed residential distance to A1 or A2 roads with E-wave velocity (Figure S1), log isovolumic relaxation time (Figure S2), LA diameter index (Figure S3), and log PASP (Figure S4) were approximately linear.

## 4. Discussion

We did not observe associations between residential distance to A1 or A2 roads and the following indicators of cardiac function: depressed LVEF, E-wave velocity, isovolumic relaxation time, LA diameter index, and PASP. Similarly to our results, Van Hee *et al.* found no association between LVEF and residential distance to the nearest major roadway [9]. We did observe a slightly increased log PASP, a marker of pulmonary vascular function, among those who lived 300–999 m from A1 or A2 roads compared to those who lived ≥1000 m from A1 or A2 roads but not among those who lived nearest to major roadways, possibly due to few people in this exposure category. Similarly, we observed a slightly smaller LA diameter index, a marker of diastolic function among those who lived 150–299 m from A1 or A2 roads, but not among those who lived <150 m. These associations may be spurious, but further research on this topic is needed to clarify the impact of traffic-related air pollution on pulmonary vascular and diastolic function. Indeed, surprisingly few prior studies have considered the possible effects of any air pollution on pulmonary vascular or diastolic function.

In sensitivity analyses, we observed that those who live 300−999 m from an A1 road may be at decreased odds of depressed LVEF. Again, this association may be spurious, but it is possible that A1 roads affect cardiac function differently than A2 roads due to traffic type and frequency. Among those without CVD, LA diameter index was positively associated with log-transformed distance to roads; this is also likely to be a spurious association. Literature demonstrates associations between air pollution exposure and risk of heart failure [4,5,9,10], but the mechanisms have yet to be fully elucidated. Few studies have examined the associations between these markers of cardiac function and exposure to traffic-related pollution. Further such studies are necessary to understand the mechanism between air pollution and heart failure, particularly in a high-risk population. It is possible that air pollution influences other aspects of cardiac structure and function. For instance, Leary *et al.* observed associations between NO_2_ concentrations and right ventricular mass and end-diastolic volume [10]. We will examine associations between residential distance to major roadways and cardiac structure in a forthcoming paper.

This study has several important limitations. First, few people (*n* = 106) resided within 150 m of an A1 or A2 road. This may have reduced our power to observe associations between residential distance to major roadways and markers of cardiac function. It is possible that those living closest to major roadways are more likely to be at higher risk of cardiovascular disease [9]. Specifically, in this study, those who resided <150 m from an A1 or A2 road were more likely to be current smokers and have poorer socioeconomic indicators compared to those who resided ≥1000 m from an A1 or A2 road. Although we adjusted for many confounders, it is possible that residual confounding exists in our models, although one would have to posit that such confounding masked the presence of an underlying true effect by biasing our associational estimates towards the null. Second, many participants were missing echocardiographic data. Specifically, a high proportion of participants were missing data on PASP (35.2%), so the results for PASP may not be generalizable. Participants who were missing some or all echocardiographic data were less educated and more likely to drink or smoke. They may have had poorer cardiovascular health, so it is possible that this source of missing data may have biased our results towards the null. Third, our study used distance to road as an indicator of traffic-related pollution. Although distance to road is an established surrogate of long-term exposure to traffic-related noise and air pollution, the potential misclassification of exposure may have biased our results towards the null. Industrial sources of pollution may have contributed to air pollution exposure and may have a different chemical composition (for instance, more nitrous acid) and thus, different physiological effects, compared to traffic sources [18]. Distance to road is a commonly used proxy measure for air pollution exposure [6,9,19,20,21,45], making our analysis comparable to other studies. Further, traffic sources account for much of the ambient air pollution in Jackson [18]. Nonetheless, this is the first study to evaluate the associations between residential proximity to major roadways and indicators of cardiac function in a large, entirely African-American population.

## 5. Conclusions

In this study of traffic-related pollution and cardiac function in an African-American population, we did not observe associations between residential distance to A1 or A2 roads and cardiac function. We did not observe evidence of how air pollution may influence cardiac function and/or heart failure. Further research is needed, particularly among different racial/ethnic groups, to elucidate this mechanism with more clarity.

## Figures and Tables

**Table 1:** Descriptive characteristics and markers of cardiac function among participants in the Jackson Heart Study by distance to A1 or A2 roads at baseline (*n* = 4866).

Characteristic	<150 m (*n* = 106)	150–299 m (*n* = 159)	300–999 m (*n* = 1161)	≥1000 m (*n* = 3440)
	Mean (SD) or %	Mean (SD) or %	Mean (SD) or %	Mean (SD) or %
Age, years, mean (SD) *	55.8 (13.0)	58.9 (12.1)	54.7 (12.1)	55.6 (12.7)
Female	67.9	64.8	62.2	64.1
BMI (kg/m^2^), mean (SD)	33.0 (8.4)	30.9 (6.7)	31.6 (7.0)	31.7 (7.2)
Highest level of education completed *				
Less than high school	33.3	21.4	16.0	20.7
High school/GED	31.4	33.3	34.1	38.4
College degree/certificate	23.8	22.0	29.7	26.3
Graduate/professional school	11.4	23.3	20.3	14.6
Household income status *				
Low	20.5	6.8	12.4	16.4
Lower-Middle	25.6	26.5	19.4	25.7
Upper-middle	20.5	29.6	30.6	30.1
High	33.3	37.1	37.6	27.8
Neighborhood SES z-score, mean (SD) ^a,^*	0.6 (6.4)	1.6 (6.6)	0.8 (5.1)	−0.8 (4.7)
Medical Insurance *				
None	13.2	12.0	12.9	13.6
Private	43.4	50.0	55.4	49.5
Public	27.4	20.9	17.3	22.7
Public and Private	16.0	17.1	14.4	14.2
Smoking status				
Never	64.2	74.8	69.4	67.5
Former	15.1	15.7	19.1	19.3
Current	20.8	9.4	11.6	13.2
Physical activity **^b^**				
Poor	49.1	48.4	46.4	50.5
Intermediate	27.4	31.5	34.2	30.8
Ideal	23.6	20.1	19.2	18.7
Alcohol consumption, past 12 months	38.8	38.2	47.2	43.7
Occupation *				
Management/professional	27.4	42.1	42.3	33.6
Service	25.5	23.3	23.0	25.7
Sales	18.9	15.1	15.1	17.8
Other	28.3	19.5	19.5	22.9
Hypertension *	55.8	67.9	57.6	62.4
Diabetes	16.0	22.3	21.6	22.3
Hyperlipidemia	33.7	29.3	29.4	29.3
Depressed LVEF (<50%)	3.8	3.8	2.6	3.9
E-wave velocity (m/s)	0.8 (0.2)	0.8 (0.2)	0.8 (0.2)	0.8 (0.2)
Isovolumic relaxation time (msec)	97.8 (22.7)	96.8 (20.0)	93.9 (21.7)	94.9 (22.3)
LA diameter index, mm/m^2^	17.7 (2.7)	17.5 (2.7)	17.6 (2.4)	17.8 (2.5)
PASP (mmHg)	27.8 (6.2)	29.0 (7.9)	28.0 (7.6)	27.8 (7.3)

Percent missing: BMI (0.1), education level (0.3), household income (15.4), medical insurance (0.4), smoking status (0.7), physical activity (0.06), alcohol consumption (2.6), occupation (0.06), hypertension (0.08), diabetes (1.1), hyperlipidemia (1.4), E-wave velocity (6.7), isovolumic relaxation time (7.8), LA diameter index (0.8), PASP (35.2); **^a^** Neighborhood socioeconomic status determined using methods described by Dubowitz [34]; **^b^** Physical activity as described in the American Heart Association’s Life’s Simple 7 [30,31]; * *p* < 0.05 (chi-square or ANOVA).

**Table 2:** Results from linear or logistic regression of residential distance to A1 or A2 roads (<150 m, 150–299 m, 300–999 m, ≥1000 m, continuous) on markers of cardiac function among participants in the Jackson Heart Study (*n* = 4866) **^a^**.

Marker of Cardiac Function	<150 m (*n* = 106)	150–299 m (*n* = 159)	300–999 m (*n* = 1161)	≥1000 m (*n* = 3440)	Log-Transformed Distance to Road (Continuous)
	Beta (95% CI) or OR (95% CI)	Beta (95% CI) or OR (95% CI)	Beta (95% CI) or OR (95% CI)	Beta (95% CI) or OR (95% CI)	Beta (95% CI) or OR (95% CI)
Depressed LVEF, OR (95% CI)	0.96 (0.34, 2.68)	0.95 (0.41, 2.22)	0.68 (0.45, 1.03)	1.0 (ref)	1.08 (0.92, 1.27)
E-wave velocity (m/s)	−0.005 (−0.04, 0.03)	0.001 (−0.03, 0.03)	0.002 (−0.01, 0.02)	0 (ref)	0.0002 (−0.005, 0.006)
Log Isovolumic relaxation time	0.03 (−0.02, 0.08)	0.01 (−0.02, 0.05)	−0.01 (−0.03, 0.006)	0 (ref)	0.0006 (−0.006, 0.007)
LA diameter index, mm/m^2^	0.06 (−0.4, 0.5)	−0.4 (−0.7, −0.02) *	−0.1 (−0.3, 0.04)	0 (ref)	0.06 (−0.0008, 0.1)
Log PASP	−0.0007 (−0.06, 0.05)	0.02 (−0.03, 0.07)	0.02 (0.001, 0.04) *	0 (ref)	−0.003 (−0.01, 0.006)

^a^ Adjusted for age, sex, BMI, alcohol consumption, physical activity status, educational level, occupation, smoking status, and neighborhood socioeconomic status; * *p* < 0.05.

## Supplementary Materials

The following are available online at www.mdpi.com//1660-4601/13/6/581/s1, Table S1: Results from linear or logistic regression of residential distance to A1 roads on markers of cardiac function among participants in the Jackson Heart Study; Table S2: Results from linear or logistic regression of residential distance to A1 or A2 roads on markers of cardiac function among participants in the Jackson Heart Study, excluding those with CVD; Figure S1. Association between E-wave velocity and natural log of residential distance to A1 or A2 roads among participants in the Jackson Heart Study, fitted using a natural spline with 3 degrees of freedom for distance to A1 or A2, adjusting for covariates; Figure S2: Association between LA diameter index and natural log of residential distance to A1 or A2 roads among participants in the Jackson Heart Study, fitted using a natural spline with 3 degrees of freedom for distance to A1 or A2, adjusting for covariates; Figure S3; Association between natural log isovolumic relaxation time and natural log of residential distance to A1 or A2 roads among participants in the Jackson Heart Study, fitted using a natural spline with 3 degrees of freedom for distance to A1 or A2, adjusting for covariates; Figure S4: Association between natural log PASP and natural log of residential distance to A1 or A2 roads among participants in the Jackson Heart Study, fitted using a natural spline with 3 degrees of freedom for distance to A1 or A2, adjusting for covariates. Shaded are represents 95% confidence interval.

## Abbreviations

The following abbreviations are used in this manuscript:

CVD	Cardiovascular disease
JHS	Jackson Heart Study
HF	Heart failure
LVEF	Left ventricular ejection fraction
LV	Left ventricular
LA	Left atrial
PASP	Pulmonary artery systolic pressure
BMI	Body mass index
NSES	Neighborhood socioeconomic status
