# Supplementary material for: Residential Proximity to Major Roadways Is Not Associated with Cardiac Function in African Americans: Results from the Jackson Heart Study
Source: Int J Environ Res Public Health. 2016 Jun 13;13(6):581. doi: 10.3390/ijerph13060581 (PMC4924038; doi:10.3390/ijerph13060581)
Supplement: Supplementary file 1 [file ijerph-13-00581-s001.pdf]

Supplementary Materials: Residential Proximity to Major Roadways Is Not Associated with Cardiac Function in African Americans: Results from the Jackson Heart Study

Anne M. Weaver, Gregory A. Wellenius, Wen-Chih Wu, DeMarc A. Hickson, Masoor Kamalesh and Yi Wang

Table S1. Results from linear or logistic regression of residential distance to A1 roads on markers of cardiac function among participants in the Jackson Heart Study (n = 4866) [a].

Marker of Cardiac Function	<150 m (n = 106)	150–299 m (n = 159)	300–999 m (n = 1161)	≥1000 m (n = 3440)	Log-Transformed Distance to Road (Continuous)
	Beta (95% CI) or OR (95% CI)	Beta (95% CI) or OR (95% CI)	Beta (95% CI) or OR (95% CI)	Beta (95% CI) or OR (95% CI)	Beta (95% CI) or OR (95% CI)
Depressed LVEF, OR (95% CI)	2.39 (0.69, 8.23)	1.47 (0.58, 3.73)	0.49 (0.28, 0.86) *	1.0 (ref)	1.07 (0.90, 1.28)
E-wave velocity (m/s)	−0.04 (−0.1, 0.03)	−0.007 (−0.05, 0.03)	0.0009 (−0.01, 0.02)	0 (ref)	−0.003 (−0.009, 0.004)
Log isovolumic relaxation time	0.02 (−0.06, 0.1)	0.03 (−0.02, 0.08)	−0.006 (-0.02, 0.01)	0 (ref)	0.007 (−0.0005, 0.01)
LA diameter index, mm/m²	0.2 (−0.5, 1.0)	−0.1 (−0.6, 0.4)	−0.1 (−0.3, 0.03)	0 (ref)	0.06 (−0.01, 0.1)
Log PASP	0.02 (−0.07, 0.1)	0.04 (−0.02, 0.1)	0.006 (−0.2, 0.03)	0 (ref)	−0.007 (−0.02, 0.003)

[a] Adjusted for age, sex, BMI, alcohol consumption, physical activity status, educational level, occupation, smoking status, and neighborhood socioeconomic status; * $p < 0.05$.

Table S2. Results from linear or logistic regression of residential distance to A1 or A2 roads on markers of cardiac function among participants in the Jackson Heart Study, excluding those with CVD (n = 4353) [a].

**Marker of Cardiac Function**	**<150 m (*n* = 106)**	**150–299 m (*n* = 159)**	**300–999 m (*n* = 1161)**	**≥1000 m (*n* = 3440)**	**Log-Transformed Distance to Road (Continuous)**
	**Beta (95% CI) or OR (95% CI)**	**Beta (95% CI) or OR (95% CI)**	**Beta (95% CI) or OR (95% CI)**	**Beta (95% CI) or OR (95% CI)**	**Beta (95% CI) or OR (95% CI)**
Depressed LVEF, OR (95% CI)	0.63 (0.15, 2.63)	0.89 (0.32, 2.50)	0.66 (0.41, 1.07)	1.0 (ref)	1.08 (0.90, 1.30)
E-wave velocity (m/s)	−0.004 (−0.04, 0.04)	0.003 (−0.03, 0.04)	0.0007 (−0.01, 0.01)	0 (ref)	0.0005 (−0.005, 0.006)
Log isovolumic relaxation time	0.02 (−0.03, 0.07)	0.02 (−0.02, 0.06)	−0.009 (−0.03, 0.007)	0 (ref)	0.0003 (−0.007, 0.007)
LA diameter index, mm/m^2	−0.03 (−0.5, 0.4)	−0.4 (−0.8, −0.03)*	−0.2 (−0.3, 0.005)	0 (ref)	0.07 (0.01, 0.1) *
Log PASP	0.002 (−0.06, 0.06)	0.03 (−0.02, 0.08)	0.02 (−0.005, 0.04)	0 (ref)	−0.002 (−0.01, 0.006)

[a] Adjusted for age, sex, BMI, alcohol consumption, physical activity status, educational level, occupation, smoking status, and neighborhood socioeconomic status; * $p < 0.05$.

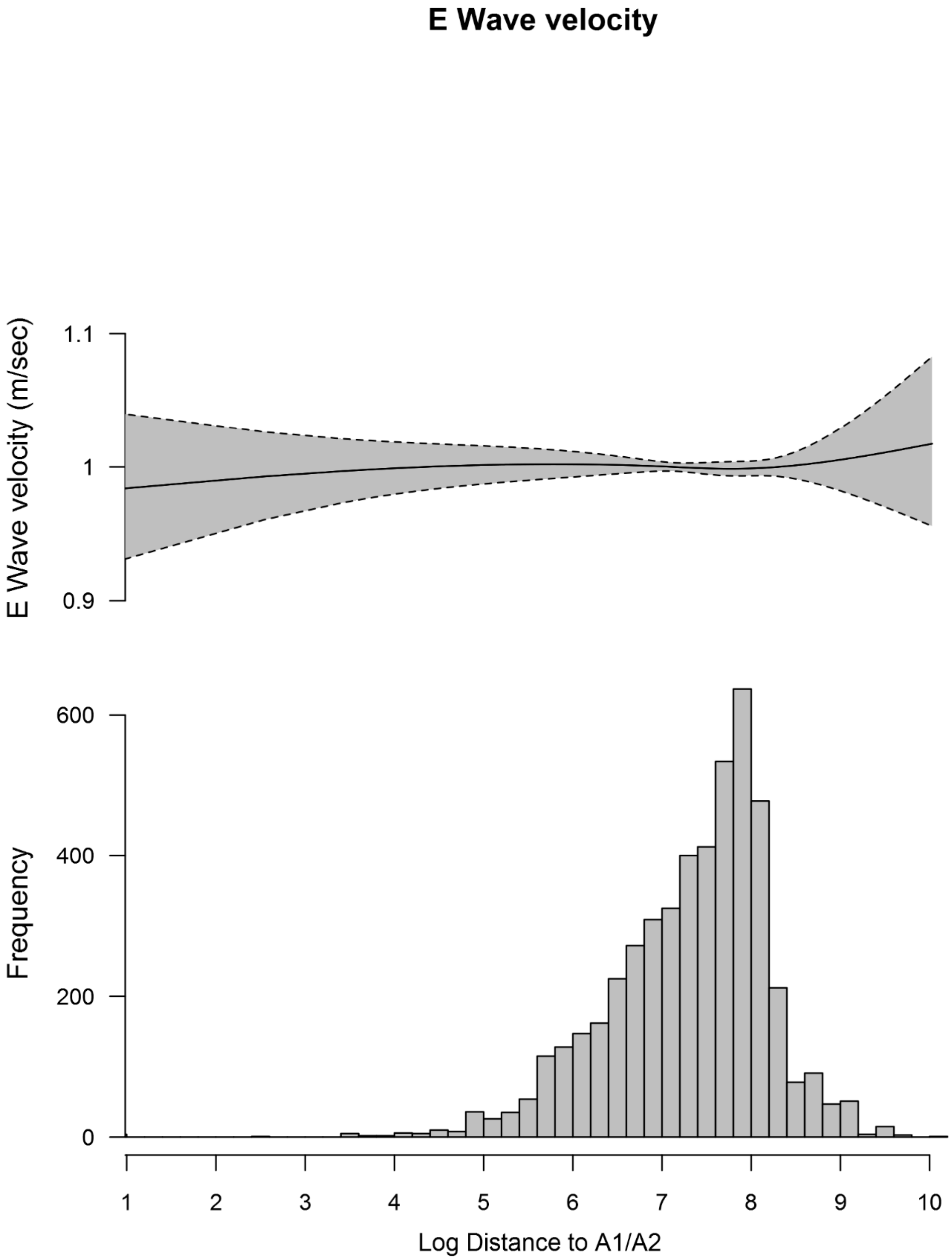

Figure S1. Association between E-wave velocity and natural log of residential distance to A1 or A2 roads among participants in the Jackson Heart Study, fitted using a natural spline with 3 degrees of freedom for distance to A1 or A2, adjusting for covariates. Shaded are represents 95% confidence interval. ($n = 4866$) [a]; [a] Adjusted for age, sex, BMI, alcohol consumption, physical activity status, educational level, occupation, smoking status, and neighborhood socioeconomic status.

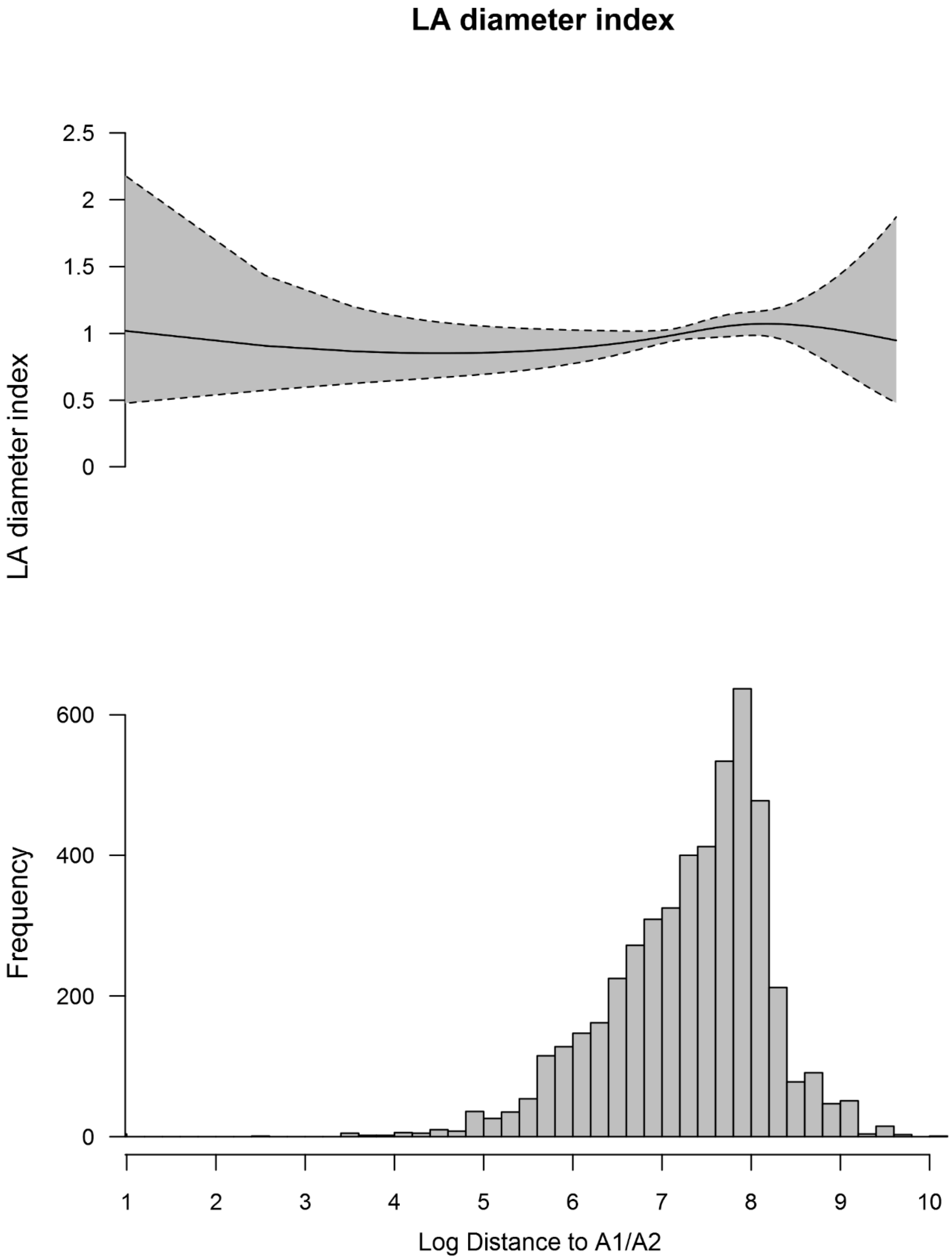

Figure S2. Association between LA diameter index and natural log of residential distance to A1 or A2 roads among participants in the Jackson Heart Study, fitted using a natural spline with 3 degrees of freedom for distance to A1 or A2, adjusting for covariates. Shaded are represents 95% confidence interval. ($n = 4866$) [a]; [a]Adjusted for age, sex, BMI, alcohol consumption, physical activity status, educational level, occupation, smoking status, and neighborhood socioeconomic status.

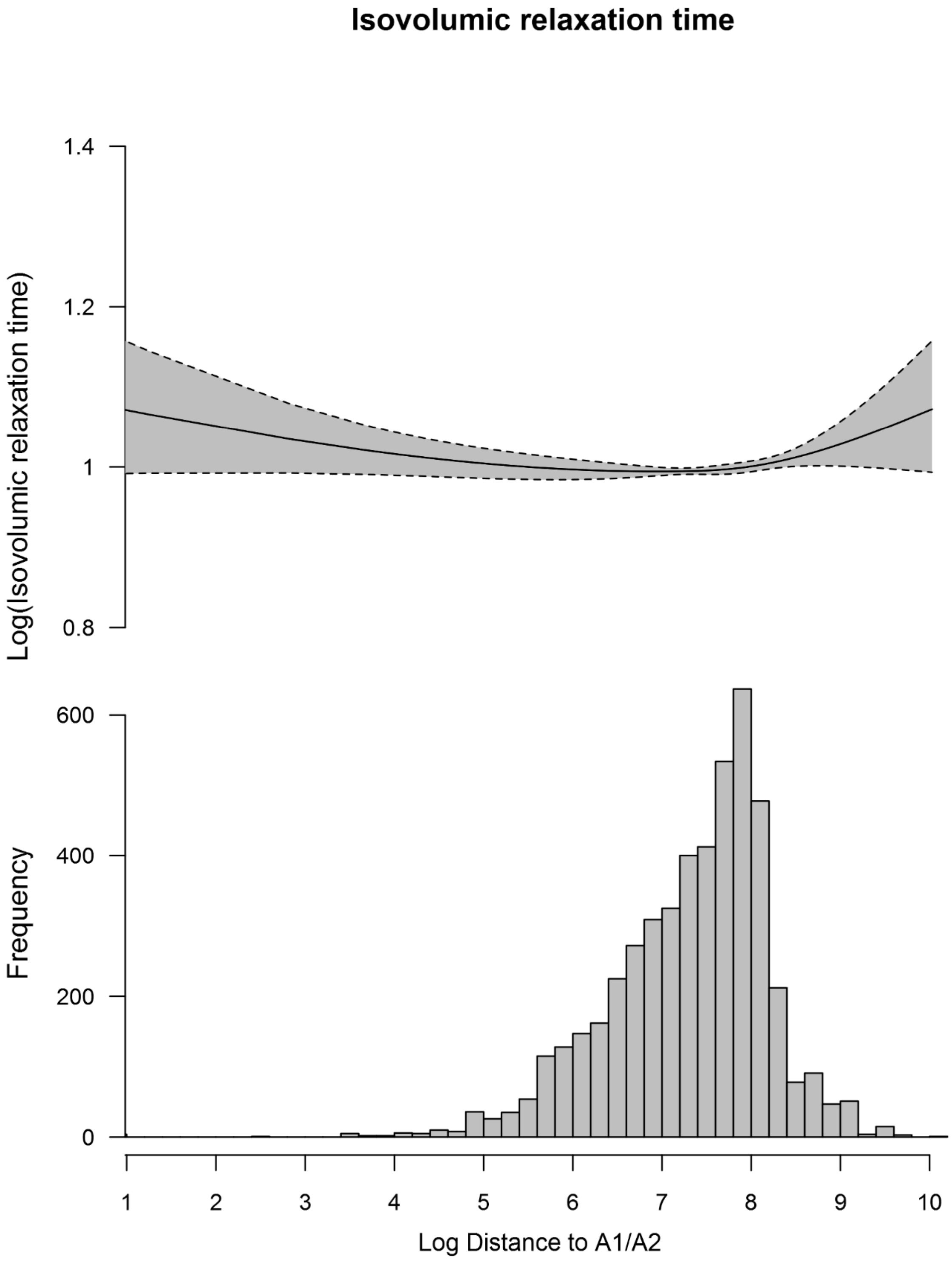

Figure S3. Association between natural log isovolumic relaxation time and natural log of residential distance to A1 or A2 roads among participants in the Jackson Heart Study, fitted using a natural spline with 3 degrees of freedom for distance to A1 or A2, adjusting for covariates. Shaded are represents 95% confidence interval. (*n* = 4866) [a]; [a] Adjusted for age, sex, BMI, alcohol consumption, physical activity status, educational level, occupation, smoking status, and neighborhood socioeconomic status.

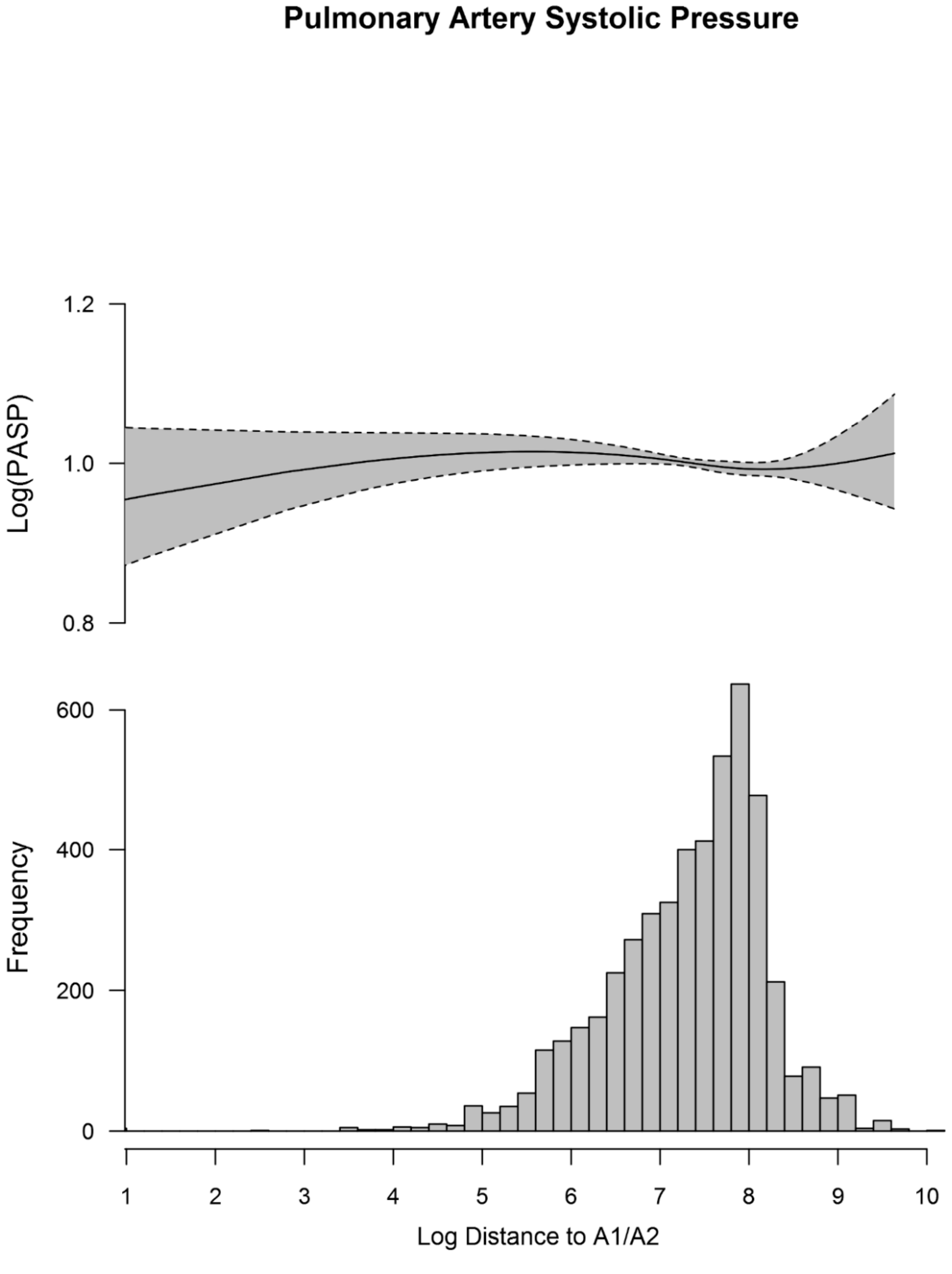

Figure S4. Association between natural log PASP and natural log of residential distance to A1 or A2 roads among participants in the Jackson Heart Study, fitted using a natural spline with 3 degrees of freedom for distance to A1 or A2, adjusting for covariates. Shaded are represents 95% confidence interval. (n = 4866) [a]; [a] Adjusted for age, sex, BMI, alcohol consumption, physical activity status, educational level, occupation, smoking status, and neighborhood socioeconomic status.

© 2016 by the authors; licensee MDPI, Basel, Switzerland. This article is an open access article distributed under the terms and conditions of the Creative Commons by Attribution (CC-BY) license (http://creativecommons.org/licenses/by/4.0/).
